# Globally distributed root endophyte *Phialocephala subalpina* links pathogenic and saprophytic lifestyles

**DOI:** 10.1186/s12864-016-3369-8

**Published:** 2016-12-09

**Authors:** Markus Schlegel, Martin Münsterkötter, Ulrich Güldener, Rémy Bruggmann, Angelo Duò, Matthieu Hainaut, Bernard Henrissat, Christian M. K. Sieber, Dirk Hoffmeister, Christoph R. Grünig

**Affiliations:** 1 Institute of Integrative Biology (IBZ), Forest Pathology and Dendrology, ETH Zürich, 8092 Zürich, Switzerland; 2 Institute of Bioinformatics and Systems Biology, Helmholtz Zentrum München, German Research Center for Environmental Health, 85764 Neuherberg, Germany; 3 Department of Genome-oriented Bioinformatics, Technische Universität München, Wissenschaftszentrum Weihenstephan, 85354 Freising, Germany; 4 Interfaculty Bioinformatics Unit and Swiss Institute of Bioinformatics, University of Berne, Baltzerstrasse 6, 3012 Bern, Switzerland; 5 Architecture et Fonction des Macromolécules Biologiques (AFMB), UMR 7257 CNRS, Université Aix-Marseille, 163 Avenue de Luminy, 13288 Marseille, France; 6 DOE Joint Genome Institute, 2800 Mitchell Drive, Walnut Creek, CA 94598 USA; 7 Friedrich-Schiller-Universität, Pharmazeutische Mikrobiologie, Winzerlaer Strasse 2, 07745 Jena, Germany; 8 Microsynth AG, Schützenstrasse 15, 9436 Balgach, Switzerland

**Keywords:** Comparative genomics, Lifestyle, Root endophyte, Species complex, Parasitism-mutualism continuum

## Abstract

**Background:**

Whereas an increasing number of pathogenic and mutualistic ascomycetous species were sequenced in the past decade, species showing a seemingly neutral association such as root endophytes received less attention. In the present study, the genome of *Phialocephala subalpina*, the most frequent species of the *Phialocephala fortinii* s.l. – *Acephala applanata* species complex, was sequenced for insight in the genome structure and gene inventory of these wide-spread root endophytes.

**Results:**

The genome of *P. subalpina* was sequenced using Roche/454 GS FLX technology and a whole genome shotgun strategy. The assembly resulted in 205 scaffolds and a genome size of 69.7 Mb. The expanded genome size in *P. subalpina* was not due to the proliferation of transposable elements or other repeats, as is the case with other ascomycetous genomes. Instead, *P. subalpina* revealed an expanded gene inventory that includes 20,173 gene models. Comparative genome analysis of *P. subalpina* with 13 ascomycetes shows that *P. subalpina* uses a versatile gene inventory including genes specific for pathogens and saprophytes. Moreover, the gene inventory for carbohydrate active enzymes (CAZymes) was expanded including genes involved in degradation of biopolymers, such as pectin, hemicellulose, cellulose and lignin.

**Conclusions:**

The analysis of a globally distributed root endophyte allowed detailed insights in the gene inventory and genome organization of a yet largely neglected group of organisms. We showed that the ubiquitous root endophyte *P. subalpina* has a broad gene inventory that links pathogenic and saprophytic lifestyles.

**Electronic supplementary material:**

The online version of this article (doi:10.1186/s12864-016-3369-8) contains supplementary material, which is available to authorized users.

## Background

Plant roots are confronted with the colonization of symbiotic fungal species ranging from pathogens to mutualists [1]. While research has largely been focused on the symbiotic and pathogenic interactions, seemingly neutral associations with plant roots by endophytes received less attention [2, 3]. Endophytic fungi colonize functional roots tissues but disease symptoms do not develop at all or at least not for prolonged periods of time [4]. Despite their prevalence in many ecosystems, little is known about the nature of interaction with their hosts [5, 6]. It is assumed that they behave along the mutualism - antagonism continuum depending on host conditions and environment, as shown for some mycorrhizal fungi [7–9].

Species belonging to the helotialean *Phialocephala fortinii* s.l. – *Acephala applanata* species complex (PAC) are the dominant root endophytes in woody plant species [5]. PAC shows a global distribution as PAC species colonize roots from arctic to subtropical plant species throughout the northern hemisphere [10–12]. Recently, a study proved the presence of PAC on the southern hemisphere [13]. In contrast to ectomycorrhizal species (EcM), which are usually confined to primary, non-lignified roots, PAC can be found in all parts of the root system, predominatly on fine roots < 3 mm where up to 80% of randomly sampled roots were colonized [5]. In addition, PAC species are belonging to the first colonizers of tree seedlings in natural forest ecosystems, infecting them within a few weeks after germination [14].

PAC is composed of more than 15 cryptic species [10], eight of which were formally described [15, 16]. Among the strains sampled from a single study site, PAC species form communities of up to 10 sympatrically occurring species. In contrast to agricultural ecosystems, PAC communities remain stable for several years [17] although minor long-term changes in the frequency of species can be observed [18]. Most PAC communities are dominated by few species and additional species occur at low frequencies [5] as observed in many other community structures of biological systems [19]. Species diversity and community structure do not correlate with the tree community, geographical location, soil properties, management practices nor does climate, precipitation and temperature [10]. Host specificity of PAC species was not observed [5] except for *A. applanata* that was almost exclusively isolated from species belonging to the Pinaceae but rarely from ericaceous roots of the ground vegetation [14].

Despite the fact that PAC species are highly successful colonizers of plant roots and widely distributed in natural ecosystems, their ecological role is still poorly understood. They were described as beneficial, neutral or pathogenic for different hosts, growing conditions and fungal strains [5, 20, 21]. New results comparing the effect of PAC species and strains on *Picea abies* indicate that the outcome of the interactions is mainly driven by the fungal genotype and follow the antagonism-mutualism continuum. Whereas some of the PAC/*P. subalpina* strains were shown to be pathogenic and killed most of the seedlings, others were benign [22]. Nevertheless, infection by PAC is costly for the plant since an increase in plant growth was never observed [22]. The outcome of PAC-host interactions depends on the ability of PAC strains to invade and colonize host root tissues. This is evident by the health status of Norway spruce seedlings, which negatively correlates with the biomass of the fungus in roots [22, 23]. However, negative effects of harmful PAC strains are reduced by the co-colonization of ectomycorrhizal fungi and other PAC strains [24].

**Fig. 1:**
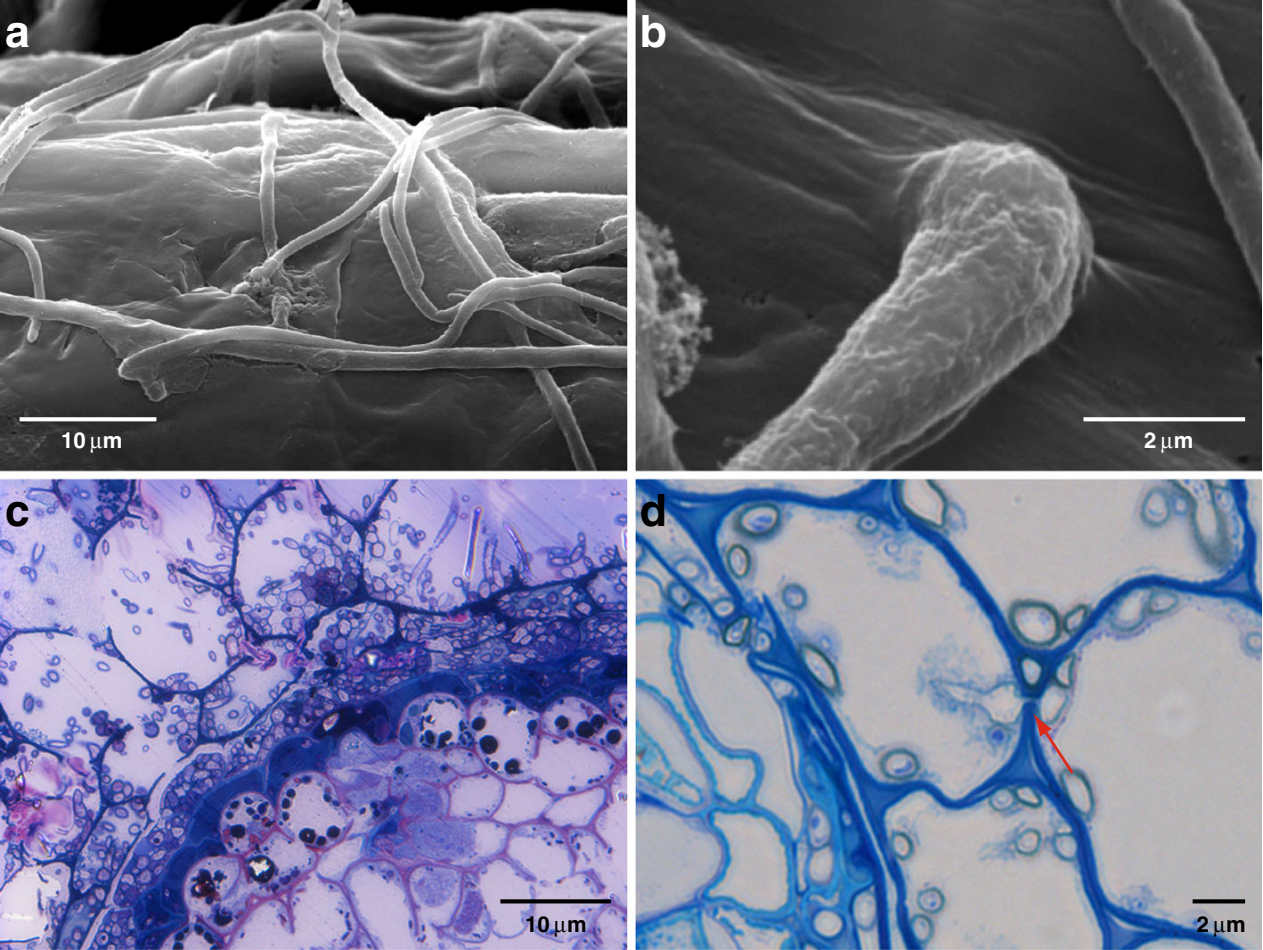
Key features of the colonization of roots by PAC species. Key steps in the colonization of roots by PAC species (V. Queloz, unpublished). Figure 1a, b. Surface colonization and appressoria/hyphopodia formation of *P. subalpina* on *Cistus incanus* roots. Figure 1c. Cross-section of *Pinus strobus* root colonized by PAC stained using borax, methylene blue and toluidine blue and counterstained with basic fuchsine. The fungus completely colonizes the cortical tissue up to the endodermis. Accumulation of phenolic compounds in the vascular cylinder is evident by the presence of dark granular structures. Figure 1d. Example of intracellular colonization of *P. subalpina* in *C. incanus* cortex (arrow)

The dynamics of PAC-host colonization was rarely studied [25–27], and data on intraspecific variation of colonization dynamics for different PAC species is missing completely. In general, PAC strains form hyphopodia- or appressoria-like structures to enter root hairs or epidermal cells (Fig. 1a, b). After entering the root, PAC strains grow inter- and intracellularly and colonize the cortex but rarely invade the vascular cylinder (Fig. 1c, d). Intercellular labyrinthine fungal tissue resembling the Hartig net in ectomycorrhizal fungi as well as mantel-like structures were occasionally observed for PAC [27–29].

Host defense reactions such as cell-wall appositions or lignituber formation were rarely observed [27]. Intracellular hyphae traverse host cells by narrow penetration hyphae without apparent lysis of the plant cell wall while the host cell cytoplasma disintegrates after colonization by PAC hyphae (Fig. 1d). Pertaining to the ultrastructural level, hyphae are not surrounded by host-derived perifungal membranes, which are regarded as hallmark for biotrophic fungal associations [27]. Finally, cortical cells of the plant are often associated with thick-walled, heavily melanized fungal cells, i.e. microsclerotia, which were shown to accumulate reserve substances [5, 25].

The availability of genomic sequences provides information on the gene inventory of species and identifies characteristic genomic structures and gene sets associated with different lifestyles [30–33]. Although the number of sequenced fungal genomes increases rapidly, genome sequencing of ascomycetes was mostly restricted to pathogenic, saprophytic or well-known mutualistic species. In contrast, only few endophytes were sequenced and restricted to endophytes in the Clavicipitaceae [34, 35]. Clavicipitalean endophytes are obligate biotrophs, colonize their hosts systemically and follow a very close symbiotic lifestyle with their hosts but roots are not colonized [36]. This sets them apart from non-clavicipitalean endophytes isolated from all parts of the plants at high frequencies. An exception is the genome of *Phialocephala scopiformis*, a foliar endophyte, for which the draft genome was recently published albeit with no analysis [37]. In the present study, the genome of *P. subalpina*, the most frequent species of the PAC, was sequenced and compared to the genomes of 13 ascomycetous species with different lifestyles to get first insights in the genome structure and gene inventory of non-clavicipitalean endophyte.

## Results

### Phialocephala subalpina holds a large and feature-rich genome

The genome of *P. subalpina* strain UAMH 11012 was sequenced at 25 x coverage using the Roche/454 GS FLX technology. Sequence data was assembled into 204 scaffolds (excluding rDNA repeat and mtDNA) with a genome size of 69.7 Mb and an average GC content of 45.9% (Table 1). The complete rRNA repeat (part of this assembly) and the mtDNA (http://www.ebi.ac.uk/ena/data/view/JN031566) [38] were assembled manually and validated by Sanger sequencing. Data sets are accessible at http://pedant.helmholtz-muenchen.de/genomes.jsp?category=fungal. The genome and annotation data was submitted to the European Nucleotide Archive (ENA, http://www.ebi.ac.uk/ena/data/view/FJOG01000001-FJOG01000205). Mapping of reads data against the assembled genome did not reveal any significant deviations from the average 25x coverage except for the mtDNA (2,629x) and rDNA repeat (1,422x) indicating that no repetitive sequences were collapsed in short scaffolds leading to an underestimation of the genome size.

**Table 1:** Genome statistics for *Phialocephala subalpina*

Genome size [Mb]	69.69
Scaffolds	205
Scaffolds N50 [kb]	1,449
N50 number of scaffolds	16
GC (%)	45.9
Predicted protein-coding genes	20,173
Average exon length [bp]	443.9
Average intron length [bp]	80.2
Average number of introns per gene	2.7
tRNAs	91
TEs content	5.70%
Other repeats^a^	8.10%

^a^tandem repeats, SSR, and low-complexity DNA

**Fig. 2:**
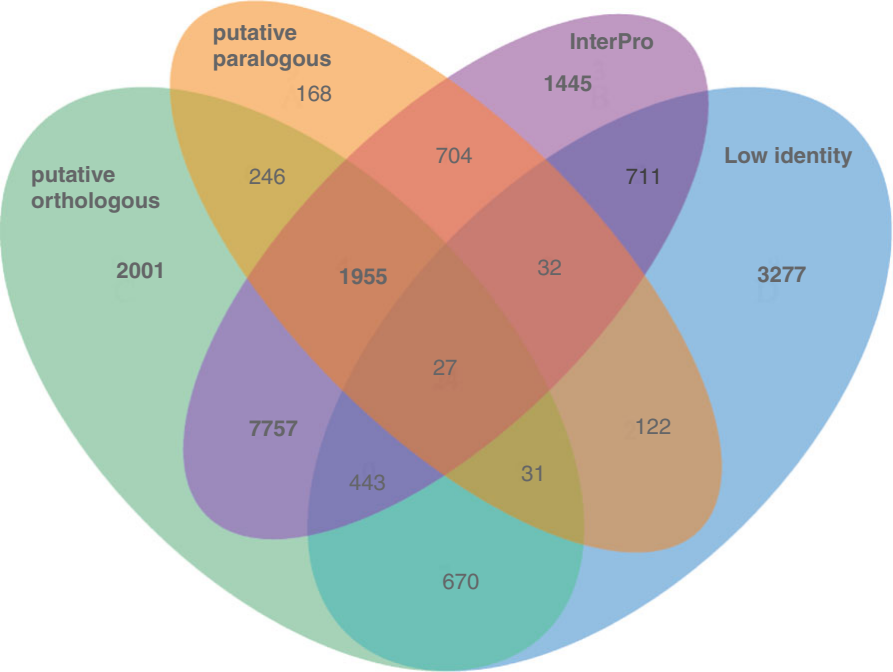
Classification of gene models of *P. subalpina.* Venn diagram showing a classification of gene models based on four different characteristics. Putative orthologous gene models were determined by QuartetS analysis including 13 ascomycetous species (see Table 2), putative paralogous gene models in *P. subalpina* were analyzed using the Uclust algorithm, low identity gene models showing <30% identity in SIMAP searches and gene models including ≥1 InterPro accessions. Five hundred eighty four single-copy and high-identity genes not including InterPro accessions were not covered by one of the four characteristics

The annotation pipeline and manual validation resulted in 20,173 gene models. In addition, 91 tRNA genes coding for all amino acids and 20 5S rRNA loci were identified. None of the expected single-copy core orthologous genes found in eukaryotes (248 and 246 genes, see material and methods) was missing in the protein predications for *P. subalpina* indicating that the core gene inventory had completely been covered. This was supported by EST data as 28,045 out of 28,092 assembled transcripts (99.83%) mapped to the assembly with high coverage (Additional file 1). 73.6% of the 20,173 proteins showed an identity >30% with known proteins in the Similarity Matrix of Proteins database (SIMAP) [39]. The remaining 5,313 proteins with less than 30% identity included 4,233 species-specific *P. subalpina* proteins. No significant length difference was observed among the high identity and the low identity genes (Additional file 2A). Moreover, mapping of the 4,233 proteins against the 454 EST dataset and RNA-Seq data showed that 2,833 of these gene models were covered by ≥50% of the ORF length with EST/RNA-Seq data (Additional file 2B). A classification scheme of the gene models based on different analysis is given in Fig. 2.

### Classification of repetitive elements

**Fig. 3:**
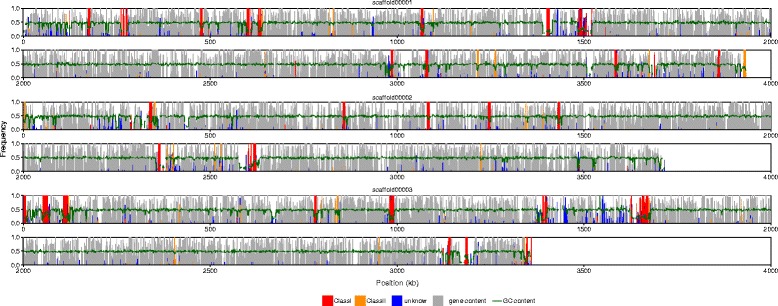
Overview of the gene content (gray), repeat content and the average GC content (green line) in selected scaffolds. The vertical lines (bars) represent the fraction of bases covered by genes and repeats within consecutive 1 kb windows

Repeats that could be classified as transposable elements (TEs) accounted for 5.7% of the genome sequence (Table 2). In contrast, 1.8% of repeats identified in RepatScout analysis could not be classified to any TE family. TE were dominated by Class I elements of the Copia and Gypsy families attributing for 55% of all TEs. In contrast, class II TEs were less abundant and were dominated by Tc1 Mariner and Helitron elements. A large fraction of the repeat consensus sequences of the RepeatScout analysis could not be assigned to any TE family. TEs of all classes/families were evenly scattered throughout the genome of *P. subalpina* and no evidence for TE-rich islands was observed (Fig. 3). Besides the TEs, the genome of *P. subalpina* also included low-complexity sequences, tandem as well as simple sequence repeats (total 8.1% of the genome).

**Table 2:** Classification and frequency of the most important transposable elements in the genome of *P. subalpina*

TE class	TE family	In % of TEs	Cumulative length in the genome [in kb]	Proportion of the genome
Class I	Gypsy	28.9%	1,452	2.1%
	Copia	26.1%	1,308	1.9%
	non LTR	4.6%	229	0.3%
Class II	Tc1 Mariner	8.2%	411	0.6%
	Helitron	3.9%	194	0.3%
	MITE	1.2%	59	0.1%
	hAT	0.6%	32	0.0%
	Mutator	0.4%	18	0.0%
Not classified	not classified	26.2%	1,284	1.8%

### Presence of RNAi pathway and evidence for RIP mechanisms

Homologs of DICER, ARGONAUT and RNA-dependent RNA polymerase genes were found in multiple copies in the genome of *P. subalpina* and comparison with other ascomycetes showed that the copy number of each of the three genes was higher than with other ascomycetes (Table 3). Similarly, several copies of cytosine methyltransferase gene of the Dnmt1 family were present in the genome of *P. subalpina* (Table 3). The cytosine methyltransferases encoded in *P. subalpina* were classified both based on the presence of InterPro domains and arrangements of the domains in comparison with *Neurospora crassa*, *Ascobolus immersus*, *Sclerotinia sclerotiorum* and *Botrytis cinerea* homologues. Whereas gene models PAC_15881 and PAC_01402 included only the C-5 cytosine methyltransferase domain (IPR001525) and showed homologies with the *RiD* gene of *N. crassa*, the other two genes (PAC_07461, PAC_02147) encoded the C-5 cytosine methyltransferase domain as well as BAH domains (IPR001025). Gene PAC_07461 has a high similarity with *N. crassa Dim2* whereas gene PAC_02147 has a high similarity with *Masc2* of *A. immersus*.

**Table 3:** Presence of RNAi and RIP core proteins in different fungal genomes

Mechanism	Gene	Ps	Om	Ssc	Bc	Bg	Nc	Ca	Sc	Sp	Median
RNAi	Argo	4	2	2	3	2(3)	2	1	0	1	2
	Dicer	4	2	2	2	2	2	0	0	1	2
	RdRP	5	3	3	3	1	3	0	0	1	3
RIP	Dnmt1 family	4	3	3	2(3)	0	2	0	0	0	1

*Ps Phialocephala subalpina*, *Om Oidiodenron maius*, *Ssc Sclerotinia sclerotiorum*, *Bc Botrytis cinerea*, *Bg Blumeria graminis*, *Nc Neurospora crassa*, *Ca Candida albicans*, *Sc Saccharomyces cerevisiae*, *Sp Schizosaccharomyces pombe*

**Fig. 4:**
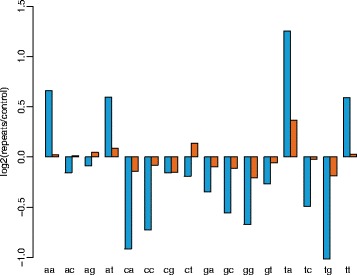
Change in dinucleotide frequencies in repeat regions. Change in the dinucleotide frequencies observed in the repeat regions of *P. subalpina* (blue) and *S. sclerotiorum* (orange) compared to genomic control regions

A clear difference in the dinucleotide frequencies was observed in repeat versus genomic control regions and the difference in dinucleotide frequencies was more pronounced in *P. subalpina* than in *S. sclerotiorum* (Fig. 4). TpA dinucleotides were significantly overrepresented in repeats whereas CpA/TpG were underrepresented suggesting a dominant mode of RIP for CpA to TpA mutations. In addition, repeat regions were generally rich in AT content as also ApA, ApT and TdT were more frequent in repeats than in non-repeat genomic regions (Fig. 3).

### The genome indicates horizontal gene transfer (HGT) events from bacteria co-occurring in the same habitat

**Fig. 5:**
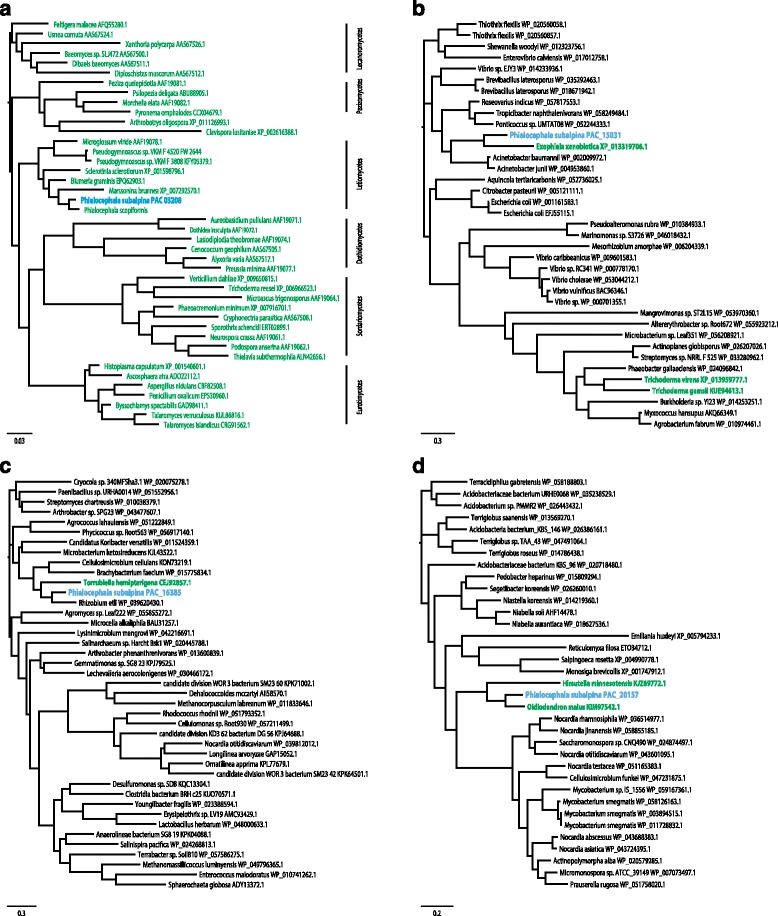
Phylogenetic analysis of genes likely acquired by horizontal gene transfer. Figure 5a. Phylogenetic tree of a conserved housekeeping gene (RPB2) of PAC and related fungi. Figure 5b-d. Phylogenetic trees of four *P. subalpina* genes likely acquired by HGT. The *P. subalpina* protein sequences cluster with bacterial proteins. Some have close (but well separated) relatives from other Ascomycetes (5c, d). Colour indications: blue = *P. subalpina*, black = non-fungal species, green = fungal species

Twenty one out of 163 genes, originally with a non-fungal best SIMAP hit, showed a skewed taxonomic distribution or higher bit scores with non-fungal taxa in BLAST searches against the NCBI non-redundant protein (nr) database (Table 4). Phylogenetic analysis for these 21 genes showed that they likely result of HGT as the gene trees significantly deviate from the expected species tree (for three examples see Fig. 5b-d; see also http://purl.org/phylo/treebase/phylows/study/TB2:S20196). In contrast, the *RPB2* gene which was used as a control in the analysis, showed the expected ascomycetous phylogeny (Fig. 5a). In the majority of HGTs, the protein sequences taken from the phylogenetically closest non-fungal species were derived from soil-borne bacteria (i.e*. Collimonas arenae* or *Brevibacillus laterosporus*) or to live in the rhizosphere (i.e. *Frankia* sp.) and share therefore the same habitat as *P. subalpina*. In 12 of the phylogenies, one or few of the closest relatives were of fungal origin while most of the remaining species were of prokaryotic origin. In several cases, *P. subalpina* clusters together with *O. maius* and/or *P. scopiformis* (Table 4 & Fig. 5d).

**Table 4:** Genes of *P. subalpina* likely acquired by horizontal gene transfer from non-fungal species

Protein	Putative function	SignalP	Remarks	Order	Closest species	Habitat
PAC_13705	fatty acid desaturase			n.a.	*Sphaerosoma arcticus*	n.a.
PAC_15031	metallo-β-lactamase			*Bacillales*	*Brevibacillus laterosporus*	soil/water/insects
PAC_18575				*Acidobacteriales*	*Acidobacteria* bacterium KBS 146	soil
PAC_13085	β-lactamase			*Caulobacteriales*	*Phenylobacterium* sp.	plant root associated
PAC_16385	terpenoid cyclase			*Rhizobiales*	*Chelativorans* sp.	soil/rhizosphere
PAC_19778	glyoxalase		gene fragment	*Burkholderiales*	*Collimonas arenae*	soil
PAC_12936	glycoside hydrolase		with *P. scopiformis*	*Burkholderiales*	*Paraburkholderia* sp.	plant-associated bacteria
PAC_20157	hydrolase	secreted protein	with *O. maius*	*Sphingobacteriales*	*Pedobacter heparinus*	soil
PAC_01946	adenine deaminase		with P. scopiformis	*Actinomycetales*	*Frankia* sp. EuI1c	soil/plant symbiont
PAC_03408	kynurenine formamidase			*Actinomycetales*	*Frankia* sp. EUN1f	soil/plant symbiont
PAC_03301		secreted protein	with *O. maius and P. scopiformis*	*Actinomycetales*	*Streptomyces* sp.	mostly soil
PAC_17397	methyltransferase		with *O. maius*	*Actinomycetales*	*Streptomyces* sp.	mostly soil
PAC_19296				*Actinomycetales*	*Streptomyces* sp.	mostly soil
PAC_14321			with *O. maius*	*Burkholderiales*	*Curvibacter lanceolatus*	n.a.
PAC_07909	quinoprotein amine dehydrogenase	secreted protein	with *O. maius*	*Burkholderiales*	*Paraburkholderia udeis*	soil/rhizosphere
PAC_06755	carotenoid oxygenase		with *O. maius*	*Actinomycetales*	*Streptomyces* sp.	mostly soil
PAC_02359				*Actinomycetales*	*Mycobacterium avium*	water/soil
PAC_18364	peptidase		with *P. scopiformis*	*Rhizobiales*	*Bradyrhizobium* sp.	soil/plant symbiont
PAC_05940	oleate hydratase		with *P. scopiformis*	*Spirochaetales*	*Leptospira* sp.	n.a.
PAC_15362	lipase		with *P. scopiformis*	*Actinomycetales*	various species	n.a.
PAC_11525			with *P. scopiformis*	*Ktedonobacterales*	*Ktedonobacter racemifer*	soil

### Key secondary metabolite genes

The genome of *P. subalpina* encodes a large number of genes coding for putative secondary metabolite (SM) key enzymes (Table 5). 75.8% of the SM key genes in *P. subalpina* clustered with putative orthologous genes in the other 13 species without any obvious dominance of the closely related Leotiomycete species, i.e., *P. subalpina* shared most orthologous clusters with *Aspergillus flavus*. Numerous SM key genes are clustered in putative secondary metabolite loci including genes for acyl-, and methyltransferases, oxidoreductases, cytochrome P450 monooxygenases, or transcription factors (Table 5 & Additional file 3).

**Table 5:** Key *secondary metabolite genes found in the genome of P. subalpina*

Class	Type	Gene code	SM cluster^a^	Length (aa)	Domain structure^b^	Remarks
NRPS-like	L-alpha-aminoadipate reductase	PAC_02750		1167	A-T-R	Lys2, L-lysine biosynthesis
NRPS-like	adenylating reductase	PAC_02944	8	1049	A-T-R	
NRPS-like	adenylating reductase	PAC_01324	3	1047	A-T-R	
NRPS-like	adenylating reductase	PAC_02391	6	1023	A-T-R	
NRPS-like	adenylating reductase	PAC_03877	10	1117	A-T-R	
NRPS-like	adenylating reductase	PAC_04959	12	1038	A-T-R	
NRPS-like	adenylating reductase	PAC_05733		1008	A-T-R	
NRPS-like	adenylating reductase	PAC_07003		1054	A-T-R	
NRPS-like	adenylating reductase	PAC_12811		1054	A-T-R	
NRPS-like	adenylating reductase	PAC_17490		1065	A-T-R	
NRPS-like	adenylating reductase	PAC_19780		1050	A-T-R	
NRPS-like	adenylating reductase	PAC_02202	5	1227	A-T-R-Kinase	
NRPS-like	adenylating reductase	PAC_08842		1345	A-T-R-KR	
NRPS-like	furanone synthetase	PAC_08910	17	1013	A-T-TE	
NRPS-like	quinone synthetase	PAC_14584		979	A-T-TE	
NRPS-like	unknown	PAC_19640		1394	A-T-DUF	
NRPS-like	unknown	PAC_12359		1655	A-T-DUF	
NRPS		PAC_05248	13	4676	A-T-C-A-T-C-A-T-C-T-C-T-C	Siderophore biosynthesis (Ferrichrome-like)
NRPS		PAC_13158		4838	A-T-C-A-T-C-T-C-A-T-C-T-C-T-C	Siderophore biosynthesis (Ferrichrome-like)
NRPS		PAC_15746		1639	A-T-C-T-C	
NRPS		PAC_20134		4692	C-A-T-C-A-T-C-A-T-C-A-T-C-T	
NRPS		PAC_16560		565	A-T-C	
NRPS		PAC_16746		1207	A-T-C	
NRPS		PAC_19282		1220	A-T-C	
PKS-NRPS		PAC_02326		3974	KS-AT-DH-MT-KR-T-C-A-T-R	
PKS-NRPS		PAC_08246	16	3965	KS-AT-DH-MT-KR-T-C-A-T-R	
Type I PKS	non-reducing PKS	PAC_01338	4	2542	SAT-KS-AT-PT-T-MT-TE	Citrinin biosynthesis-like
Type I PKS	non-reducing PKS	PAC_02435	7	2498	SAT-KS-AT-PT-T-MT-TE	Citrinin biosynthesis-like
Type I PKS	non-reducing PKS	PAC_03302		2223	KS-AT-DH-T-TE	
Type I PKS	non-reducing PKS	PAC_03589	9	1670	KS-AT-PT-T-TE	
Type I PKS	non-reducing PKS	PAC_07895	15	2171	SAT-KS-AT-PT-T-T-TE	Likely melanin biosynthesis
Type I PKS	non-reducing PKS	PAC_08751		2099	SAT-KS-AT-PT-T-T-TE	
Type I PKS	non-reducing PKS	PAC_10081		2125	SAT-KS-AT-PT-T-T-TE	
Type I PKS	non-reducing PKS	PAC_11435		2169	SAT-KS-AT-PT-T-T-TE	Likely melanin biosynthesis
Type I PKS	reducing PKS	PAC_00199	1	2694	KS-AT-DH-MT-ER-KR-T	Alternapyrone biosynthesis-like
Type I PKS	reducing PKS	PAC_00310	2	2283	KS-AT-DH-ER-KR-T	
Type I PKS	reducing PKS	PAC_01646		1687	AT-DH-MT-ER-KR-T	
Type I PKS	reducing PKS	PAC_04883	11	2239	KS-AT-DH-ER-KR-T	
Type I PKS	reducing PKS	PAC_06141		2258	KS-AT-DH-ER-KR-T	
Type I PKS	reducing PKS	PAC_10762		3173	KS-AT-DH-MT-ER-KR-T-Acyltransferase	
Type I PKS	reducing PKS	PAC_11350		3203	KS-AT-DH-MT-ER-KR-T-Acyltransferase	
Type I PKS	reducing PKS	PAC_14253		2590	KS-AT-DH-MT-ER-KR-T	
Type I PKS	reducing PKS	PAC_14645		2274	KS-AT-DH-ER-KR-T	
Type I PKS	reducing PKS	PAC_16276		3140	KS-AT-DH-MT-ER-KR-T-Acyltransferase	
Type I PKS	reducing PKS	PAC_17799		2411	KS-AT-DH-ER-KR-T	Lovastatin-diketide synthase-like
Type I PKS	reducing PKS	PAC_19082		2581	KS-AT-DH-MT-ER-KR-T	
Type I PKS	reducing PKS	PAC_19990		2970	KS-AT-DH-MT-KR-T-C	Lovastatin-nonaketide synthase-like
Type I PKS	reducing PKS	PAC_10712		1430	KS-AT-TH-KR-T	6-Methylsalicylate synthase-like PKS
Type III PKS		PAC_02116		479	IPR012328/IPR001099	
DMATS		PAC_15749		538	IPR017795	
Terpene		PAC_05884		483		
Terpene		PAC_13844		593	IPR017825/IPR017825/IPR002060	Squalene synthase-like
Terpene		PAC_15298		557		
Terpene		PAC_01018		338	IPR008949	
Terpene		PAC_04028		331	IPR008949	
Terpene		PAC_11164		466	IPR008949	
Terpene		PAC_12198		392	IPR008949	
Terpene		PAC_16221		336	IPR008949	

^a^ for details on the putative SM gene clusters see Additional file 3

^b^ Abbreviations for domains: *A* adenylation, *AT* acyltransferase, *C* condensation, *DH* dehydratase, *DUF* terminal NRPS domain of unknown function, *ER* enoyl reductase, *KR* ketoreductase, *KS* ketosynthase, *MT* methyltransferase, *PT* product template, *R* reduction, *SAT* starter unit-ACP-transacylase, *T* thiolation (=acyl- or aryl- or peptidylcarrier protein), *TE* thioesterase, *TH* thiohydrolase

Among the eight genes for non-reducing Type I PKSs, two gene clusters were identified in *P. subalpina* probably involved in the melanin synthesis pathway. Whereas PAC_11435 was placed in the same QuartetS cluster as *G. lozoyensis PKS1*, PAC_07895 showed the highest similarity with the gene of *A. alternata* encoding *alm* and the gene of *A. fumigatus* encoding *Alb1/PksP*. In addition, PAC_07895 was placed in a second QuartetS cluster. Adjacent to both PKS genes, a putative hydroxynaphthalene reductase gene was found (PAC_11432, PAC_07896). However, the two putative scytalone dehydratases (PAC_18365, PAC_19872) in the *P. subalpina* genome were not located within either cluster. Presence of putative orthologous genes for the two PKS for the 13 ascomycetous species included in the comparative analysis (Table 6) showed that only melanized species were included in these two clusters. Further, genes of other Leotiomycete species, such as *S. sclerotiorum*, *B. cinerea* and *M. brunnea,* were represented in both clusters as *P. subalpina*. Two NRPS genes were identified that are likely encoding siderophore synthetases (PAC_05248 and PAC_13158). Besides key enzymes in secondary metabolism, the genome of *P. subalpina* also encodes a fucose-specific lectin (PAC_07514) with similarities to the AAL protein of *Aleuria aurantia* that was shown to protect the fungus against predators and parasites [40].

**Table 6:** Species included in the comparative genomic analysis

Species	Code	Lifestyle	Class	Order	Family	Reference
*Botrytis cinerea*	Bc	Pathogen (necrotroph)	Leotiomycetes	Helotiales	Sclerotiniaceae	[55]
*Sclerotinia sclerotiorum*	Ssc	Pathogen (necrotroph)	Leotiomycetes	Helotiales	Sclerotiniaceae	[55]
*Blumeria graminis*	Bg	Pathogen (obligate biotroph)	Leotiomycetes	Erysiphales	Erysiphaceae	[124]
*Marssonina brunnea*	Mb	Pathogen (hemi-biotroph)	Leotiomycetes	Helotiales	Dermateaceae	[125]
*Fusarium oxysporum* f. sp. *lycopersici*	Fo	Pathogen (hemi-biotroph)	Sordariomycetes	Hypocreales	Nectriaceae	[56]
*Aspergillus flavus*	Af	Saprophyte (soil & rhizosphere)	Eurotiomycetes	Eurotiales	Trichocomaceae	[126]
*Trichoderma reesei*	Tr	Saprophyte (soil)^a^	Sordariomycetes	Hypocreales	Hypocreaceae	[127]
*Chaetomium globosum*	Chg	Saprophyte (soil/plant debris)	Sordariomycetes	Sordariales	Chaetomiaceae	[128]
*Penicillium chrysogenum*	Pc	Saprophyte (soil)	Eurotiomycetes	Eurotiales	Aspergillaceae	[129]
*Glarea lozoyensis*	Gl	Saprophyte(soil) ^b^	Leotiomycetes	Helotiales	Helotiaceae	[130]
*Oidiodendron maius*	Om	Saprophyte (peat bog)/ericoid mycorrhizal	Leotiomycetes	Leotiomycetes incertae sedis	Myxotrichaceae	[32]
*Tuber melanosporum*	Tm	Ectomycorrhizae	Pezizomycetes	Pezizales	Tuberaceae	[66]
*Cenoccocum geophilum*	Ceg	Ectomycorrhizae	Dothideomycetes	Pleosporomycetidae incertae sedis	Gloniaceae	[131]
*Phialocephala subalpina*	Ps	Root endophyte	Leotiomycetes	Helotiales	Dermateaceae	this paper

^a^in contrast to other *Trichoderma* species, *T. reesei* does not show mycoparasitism

^b^ITS sequences from environmental samples often derived from soil/rhizosphere

### Comparative genome analysis proves different lifestyles and enlarged gene families in P. subalpina

**Fig. 6:**
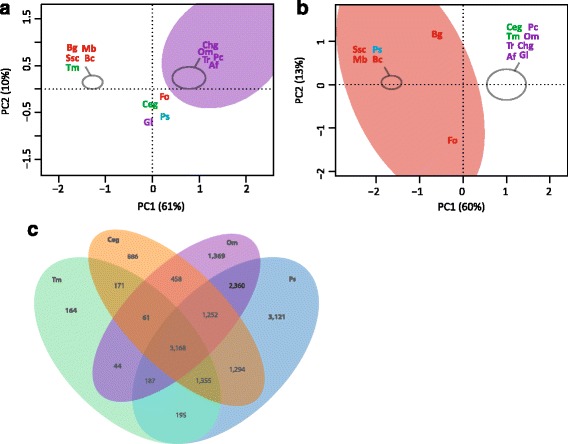
Characterization of *P.* clusters enriched for pathogens or saprophytes. Principle component analysis (PCA) based on the presence/absence of species in putative orthologous gene clusters derived from QuartetS analysis. Figure 6a. PCA analysis based on 61 gene clusters enriched for saprophytic species. Figure 6b. PCA analysis based on 163 clusters enriched in pathogens. Figure 6c. Venn diagram showing the distribution of orthologous gene clusters for the two ectomycorrhizal species *Tuber melanosporum* and *Cenococcum geophilum*, the saprophyte/ericoid mycorrhizal species *Oidiodendron maius* as well as *P. subalpina*. Abbreviations of species are given in Table 2. Color code: green: mycorrhizal species, red: plant pathogens, purple: soil saprotrophs and blue: *P. subalpina*

To explore the unexpectedly large gene set, the proteome was compared to 13 ascomycetous proteomes (Table 6) using orthologous cluster analysis, InterPro motif occurrence and a review of Carbohydrate-active enzymes (CAZymes). A total of 174,555 predicted proteins were grouped into 20,555 clusters of corresponding putative orthologous genes. Proteins of *P. subalpina* were present in 12,932 clusters (62.9%), significantly more than for any other investigated species. 1,408 clusters included proteins of all 13 species and *P. subalpina* covering core functions in the primary metabolism, energy supply and cell cycle. 163 QuartetS clusters were mostly restricted to pathogens, whereas 61 clusters were predominantly found in saprophytic species. Principal component analysis based on these two datasets showed that *P. subalpina* was either placed within the pathogens or close to the saprophytes (Fig. 6a, b). Moreover, *P. subalpina* also shared the highest number of QuartetS clusters with the two mycorrhizal species (Table 7, Fig. 6c). The most frequent FunCat annotations for the pathogen- and saprophyte-related clusters showed that the secondary and C-metabolism was most often included but also several putative orthologous proteins classified as virulence and disease factors were recognized (Table 8).

**Table 7:** Number of putative orthologous gene clusters shared by the two mycorrhizal species with the other species included in the analysis

Species	Total proteins	Total clusters	Bc	Ssc	Bg	Mb	Fo	Af	Tr	Chg	Pc	Gl	Om	Tm	Ceg	Ps
*Tm*	7,496	5,345	4,540	4,646	4,039	4,593	4,636	4,501	4,456	4,352	4,505	3,073	3,460	-	4,755	4,905
*Ceg*	14,561	8,645	5,797	5,795	4,492	5,556	6,038	5,795	5,576	5,439	5,704	3,733	4,939	4755	-	7,069

For abbreviation of species see Table 2

**Table 8:** Results of FunCat enrichment analysis for the orthologous gene clusters including *P. subalpina* that were mainly restricted to pathogenic and saprophytic species

FunCat ID^a^	Description	Pathogen-related proteins	Saprophyte-related proteins	Total proteins in QuartetS clusters	Total proteins in Ps genome
01.05	C-compound and carbohydrate metabolism	12	6	18	2473
01.20	secondary metabolism	9	7	16	2652
01.06	lipid, fatty acid and isoprenoid metabolism	7	4	11	1387
20.03	transport facilities	7	3	10	1216
11.02.03.04	transcriptional control	7	2	9	923
20.01.03	C-compound and carbohydrate transport	5	4	9	504
16.01	protein binding	6	2	8	1904
16.03.01	DNA binding	6	2	8	577
20.09.18.07	non-vesicular cellular import	5	3	8	428
32.05.05	virulence, disease factors	5	-	5	350
01.06.06.11	tetracyclic and pentacyclic triterpenes cholesterin, steroids and hopanoids metabolism	-	2	2	173

^a^see http://mips.helmholtz-muenchen.de/funcatDB/

**Fig. 7:**
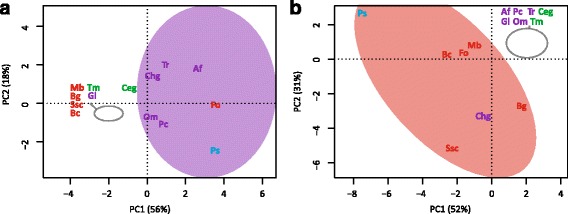
Characterization of *P. subalpina* based on overrepresented InterPro accessions for pathogens or saprophytes. Principle component analysis (PCA) based on the abundance of InterPro accessions either overrepresented in pathogenic or saprophytic species. Figure 7a. PCA analysis based on 51 InterPro accession >2x overrepresented in saprophytic species. Figure 7b. PCA analysis based on 75 InterPro accession >2x overrepresented in pathogenic species. Abbreviations of species are given in Table 2. Color code: green: mycorrhizal species, red: plant pathogens, purple: soil saprotrophs and blue: *P. subalpina*

A total of 6,556 InterPro accessions were annotated among the 14 species included in the analysis. A plateau of approx. 5,000–5,500 distinct InterPro accessions per species was observed when plotting the number of distinct InterPro accessions per species against the total number of annotated InterPro accessions per species (Additional file 4). *P. subalpina* is represented by 5,232 distinct InterPro accessions, and the highest number was observed in the two saprophyte species *T. reesei* (5,529) and *A. flavus* (5,303). In contrast, mycorrhizal species and the obligate biotrophic pathogen *B. graminis* included a significantly lower number of InterPro accessions. 963 (14.6%) InterPro accessions were significantly overrepresented in the *P. subalpina* genome compared to the average count for the other 13 ascomycetous species and 386 (5.9%) InterPro accessions were encoded >3x in the *P. subalpina* genome. Mapping of the overrepresented InterPro accessions against the gene ontology (GO) showed that catabolic/metabolic processes, transporters, and InterPro accessions involved in binding events were most frequent (Table 9). 411 (43%) of the InterPro accessions were not linked with GO annotations and *P. subalpina* included among others 534 gene models with the fungal heterokaryon incompatibility domain (IPR010730), 549 gene models with the major facilitator superfamily domain (IPR020846) and 328 gene models with ankyrin repeats (IPR020683). Moreover, several classes of CAZymes contained InterPro accessions without GO annotation such as IPR017853 (glycoside hydrolases) as well as IPR011050 (Pectin lyase fold/virulence factor) and IPR012334 (Pectin lyase fold). Only, 53 InterPro accessions were significantly underrepresented in *P. subalpina*. The underrepresented InterPro accessions frequently showed uneven distributions in the 13 ascomycetous genomes, and some of the accessions (IPR013762, IPR000477, IPR001584) are most probably related to transposable elements. Similarly to PCA based on QuartetS analysis, PCA based on InterPro accessions overrepresented in pathogenic and saprophytic species placed *P. subalpina* either in the pathogenic cluster or the saprophytic cluster (Fig. 7a, b).

**Table 9:** Enrichment of GO terms for the overrepresented InterPro accessions encoded in *P. subalpina* (each InterPro accession was only considered once per gene model)

GOID	GO description	Number of InterPro accessions	Total proteins in Ps genome
NA	no GO annotation	411	6426
GO:0055114	oxidation-reduction process	103	1138
GO:0016491	oxidoreductase activity	54	1002
GO:0005975	carbohydrate metabolic process	43	368
GO:0008152	metabolic process	38	808
GO:0003824	catalytic activity	35	733
GO:0004553	hydrolase activity, hydrolyzing O-glycosyl compounds	30	235
GO:0016020	membrane	28	459
GO:0016021	integral component of membrane	26	912
GO:0005515	protein binding	24	1065
GO:0005524	ATP binding	22	501
GO:0006508	proteolysis	22	209
GO:0055085	transmembrane transport	19	913
GO:0008270	zinc ion binding	17	882
GO:0016787	hydrolase activity	14	208
GO:0050660	flavin adenine dinucleotide binding	14	184
GO:0020037	heme binding	13	280

Enrichment of GO terms for the overrepresented InterPro accessions in *P. subalpina* compared to average number of InterPro accessions found in 13 ascomycetous genomes. Of the 963 overrepresented InterPro accessions 57% mapped to one or several GO terms and the table summarizes the GO terms with the highest numbers of distinct InterPro accessions.

**Fig. 8:**
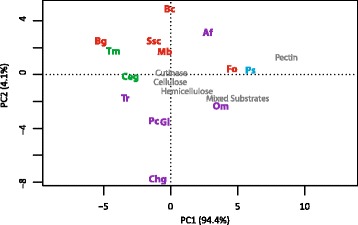
PCA analysis based on CAZyme modules involved in plant cell wall degradation (PCWDEs). Principle component analysis based on the abundance of CAZyme modules involved in cellulose, hemicellulose, pectin, and cutin breakdown. The sum of the number of CAZyme modules per compound were used for the analysis. Abbreviations of species are given in Table 2. Color code: green: mycorrhizal species, red: plant pathogens, purple: soil saprotrophs and blue: *P. subalpina*

Eight hundred eighty one gene models in *P. subalpina* were classified in 138 different CAZyme families resulting in 998 CAZyme modules, which are significantly more than observed in the other ascomycetous species. The second most frequently found CAZyme modules (883 modules) were encoded by *F. oxysporum*. The genome of *P. subalpina* was especially rich in genes coding for glycoside hydrolases (471), glycosyltransferases (150) and redox enzymes (auxiliary activities enzymes, 157). Principal component analysis based on the frequency of CAZyme modules involved in plant cell wall degradation (PCWDEs) such as cellulose, hemicellulose, pectin, cutin, and enzymes likely acting on different substrates separated *P. subalpina* from all genomes (Fig. 8). Especially modules involved in pectin breakdown were encoded in high copy numbers in the genomes of both *P. subalpina* and *F. oxysporum* but *P. sublpina* included also a higher number of modules for cellulose and hemicellulose degradation (Additional file 5). The ectomycorrhizal species *T. melanosporum* and *C. geophilum* as well as the obligate biotrophic species *B. graminis* were separated due to the small number of PCWDEs (Fig. 8).

## Discussion

In the present study we sequenced the genome of the root endophyte *Phialocephala subalpina* belonging to the *Phialocephala fortinii* s.l. – *Acephala applanata* species complex one of the most prevalent species in forest ecosystems. By comparative genomic analysis with the gene inventory of 13 other ascomycetous species we show that *P. subalpina* links pathogenic and saprophytic lifestyles.

### Genome expansion due to a large set of distinct genes in gene families

With a genome size of approximately 69.7 Mb, the *P. subalpina* genome is significantly larger than the average genome size of previously sequenced ascomycetous species [41]. Genome expansions can be caused by various events including (i) genome duplications, (ii) invasion of autonomous elements such as TEs and expansion of repetitive sequences such as microsatellites and tandem repeats into the genome, (iii) the number and length of introns and/or (iv) the expansion of the gene inventory [42, 43]. No evidence for large segmental duplications were observed in the *P. subalpina* genome by genome-wide alignments using CoCoNUT [44] and the frequency of repeats in general and TEs in special was small, considering the genome size (Additional file 6). Proliferation of TE is counteracted by three processes which act at different stages of TE proliferation. Repeat induced point mutations (RIP) act on the DNA level by introducing C to T transitions and reversing CpG to TpA dinucleotides in repeated regions [45, 46]. MIP (Methylation induced premeiotically) *de-novo* methylates repeated DNA sequences [47] and RNA interference (RNAi) suppresses TE proliferation either by heterochromatin assembly or small interfering RNAs, which silence TE transcripts [48–50]. Indirect evidence that a RIP mechanism is or was active in *P. subalpina* stems from the skewed dinucleotide distribution in repeat regions. RIP is active during the sexual cycle [51] but no sexual stage is known in *P. subalpina*. However, several lines of evidence suggest that sexual reproduction regularly occurs as (i) most PAC populations showed no gametic disequilibrium, (ii) mating types do not deviate from a 1:1 ratio in PAC populations and (iii) strong purifying selection was recorded in the mating type loci [52]. Moreover, teleomorphs are known for phylogenetically closely related species such as *Phaeomollisia piceae* or *Mollisia* spp. [53]. Therefore, it seems likely that field studies have overlooked the teleomorph of PAC species so far [52]. Common to the RIP/MIP process is that cytosine DNA methyltransferase of the Dnmt1 family play a key role [45, 54]. Homologs of cytosine DNA methyltransferases were present in *P. subalpina* including two gene models closely related to *N. crassa RiD* and one protein related to *N. crassa Dim2*. A fourth gene model found in *P. subalpina* (PAC_02147) was closest related to MASC2 of *Ascobolus immersus* which was placed in a cluster exclusively with basidiomycete species in the study of Amselem et al. [54]. However, additional analysis showed that also other Leotiomycete species included *MASC2* homologs (*B. cinerea* (CCD54489), *Sclerotinia borealis* (ESZ90943) and *M. brunnea* (XP_007291083)) adding some additional notable exceptions of ascomycetous proteins in this cluster. Beside the indirect evidence of RIP/MIP, *P. subalpina* included also RNAi key enzymes such as Dicer, Argonaute and RdRP in multiple copies. Based on these findings we hypothesize that *P. subalpina* has a well-developed arsenal of defense mechanisms in place to counteract the proliferation of TEs which may explain the comparative low frequencies of TEs.

No significant differences in intron length and the average intergenic distance were observed. However, the gene inventory was one of the largest among ascomycetes with 20,173 annotated gene models resulting in 2,500 to >10,000 more gene models than in other ascomycetes. In the light of the broad host range and the broad geographical distribution of species belonging to the *P. fortinii* s.l. – *A. applanata* species complex [1, 3, 5, 10] an enlarged gene repertoire could be expected. However, even compared to other fungal species with broad host ranges such as *S. sclerotiorum*, *B. cinerea* and *F. oxysporum* the number of gene models is large [55, 56]. The high numbers of gene models may be the result of the annotation of a large fraction of very short gene models [55] or gene models including TE fragments. In *P. subalpina*, no significant deviation in protein length distributions for low identity genes was observed and a large fraction of the low identity genes were covered by ESTs. Moreover, particular attention was paid to mask TEs. Therefore, the expansions in the gene inventory of *P. subalpina* do not result from annotation artefacts. Several factors contributed to the high number of gene models found in *P. subalpina*. First of all, *P. subalpina* showed a slightly higher fraction of putative paralogous genes than observed on average for the other 13 ascomycetous species. *F. oxysporum* showed the highest fraction of putative paralogous genes and this observation is due to large segmental genome duplication [56]. Secondly, a significant number of gene models in *P. subalpina* were species-specific as they showed no significant hits in SIMAP analysis. The high fraction of species-specific genes may be the result of the lifestyle of *P. subalpina* as well as the missing genome data of closely related species in the *Loramyces* – *Vibrissea* clade [53, 57]. Indeed, if the supposedly species-specific genes of *P. subalpina* were blasted against the recently announced genome of the closely related *P. scopiformis* [37], 28% of the gene models showed significant blast hits (>1.0 E^-14^). Thirdly, significantly more InterPro accessions were annotated in *P. subalpina* including 13,074 gene models than in any other species used for comparison. The high number of annotated InterPro accessions in the gene inventory did, however, not result in an expanded functional catalogue as the number of distinct InterPro accessions per species reached a plateau at approx. 5,200–5,500 distinct accessions indicating that “more of the same” is present in *P. subalpina*.

Our analyses show that the gene inventory of *P. subalpina* was also expanded to some extent by HGT from non-fungal organisms. A systematic analysis of the acquired prokaryotic genes by 60 fungal genomes by Marcet-Houben & Gabaldón [58] showed that species in the Pezizomycotina exhibited a high incidence of inter-domain HGT including between 4 and 63 proteins per species. The 21 HGT events found in *P. subalpina* likely represents a lower limit of events and closer inspection of uncertain candidates will likely reveal additional HGT events. Moreover, HGT events from other fungal species were shown to be another significant source for HGT [59, 60]. The phylogenetically closest protein sequences often originated from species in the Burkholderiales or Actinomycetales that colonize soil and/or roots, i.e., they share their habitat with *P. subalpina* and rendering a HGT event likely.

In almost half of the cases, the HGT event was unique or occurred recently. In contrast, also older HGT events were detected that were characterized by the presence of multiple fungal species within a clade of non-fungal species [60]. Interestingly, *O. maius* and *P. scopiformis* shared several of the 21 studied HGT events. It is possible that the HGT event occurred in a common ancestor as both fungi are related to *P. subalpina* (Fig. 6a). Alternatively, it might be possible that these genes were introduced twice independently for *O. maius* as *O. maius* can also be found in similar habitats as *P. subalpina*. It is associated with roots of ericaceous shrubs and involved in the decomposition of sphagnum peat [61]. Genes transferred by HGT can drive important evolutionary innovations as shown for plant pathogens [60, 62–64]. This was also observed in *P. subalpina* as several proteins were hydratases or peptidases. Notably, two β-lactamases were included that are involved in the detoxification of β-lactam antibiotics [65] and may result in a competitive advantage against other microbes.

### The chameleonic genome of *P. subalpina*

The ecological role of members of the *P. fortinii* s.l.-*A. applanata* species complex is still poorly understood. They were described as beneficial, neutral or pathogenic for different hosts, growing conditions and fungal strains [5, 20] and even a role in mycorrhizal associations was hypothesized [21]. In order to shed light on the lifestyle of *P. subalpina* we compared the genome against 13 genomes of other ascomycetous species with different lifestyles. All our analyses showed that the gene inventory of *P. subalpina* supports multiple lifestyles. First, *P. subalpina* shared more putative orthologous genes with the two ectomycorrhizal species included in the analysis than any other species indicating some affinities with the two species. Nevertheless, an important difference is evident. EcM fungi as well as obligate biotrophic pathogens are generally characterized by a reduced gene inventory especially for plant cell wall degrading enzymes (PCWDEs) [32]. However, *P. subalpina* encodes a high number of PCWDEs. Moreover, several EcM fungi and obligate biotrophic pathogens show genome expansions due to TE proliferation [30, 66, 67] which was absent in *P. subalpina*.

In contrast to EcM fungi, saprophytes and necrotrophic/hemibiotrophic pathogens are well endowed with enzymes involved in the degradation of plant material [32]. The main difference between these two groups is that the pathogenic species must have a specific gene inventory allowing them to invade hosts and overcome plant immune response, i.e., pathogen-associated molecular patterns (PAMP) or damage-associated molecular patterns (DMAP) [68, 69]. Whereas necrotrophic pathogens kill the host tissue by secreting effectors like toxins and/or proteins, hemibiotrophic pathogens grow intracellularly. and form specialized structures such as haustoria for nutrient uptake [70]. Recent comparative genome analysis showed that there are only few genes associated with plant pathogens that are absent in non-pathogens [33, 71]. However, domains overrepresented in necrotrophic/hemibiotrophic pathogenic species compared to saprophytes were identified [71]. When our dataset was enriched for overrepresented InterPro accessions in pathogens only few of the accessions were in accordance with Soanes et al [71]. However, a closer look showed that also of the accessions listed by Soanes et al [71] were also overrepresented in our dataset yet with a factor <2x. Irrespective of which InterPro accession list was used for analysis, *P. subalpina* was placed close to the pathogenic species (Fig. 7b and Additional file 7), indicating the robustness of the analysis. The list included genes with protease/peptidase domains, cutinases, pectinases, genes including a necrosis inducing domain or genes with chitin-binding modules. Many of these gene classes were shown to play a pivotal role in plant-pathogen interactions [68, 70]. For example, PAMP-induced host chitinases can either be overcome by the action of secreted proteases [72] or the secretion of LysM effectors that may be coupled with a chitin-binding module [73, 74]. The finding that *P. subalpina* includes the repertoire of pathogenic species fits well with the recent host-fungus interactions studies showing that PAC strains behave along the antagonism-mutualism continuum and are localized rather towards antagonistic interactions as the colonization results in reduced biomass accumulation of the host [22]. However, strong strain-specific differences were observed in the outcome of the interaction with some strains killing the majority of the seedlings and others only marginally affecting the host [22] and future studies are needed to understand the underlying mechanisms. Despite the pathogenic gene repertoire observed in *P. subalpina*, host defense mechanisms such as lignituber formation and cell wall appositions are rarely observed during the invasion of PAC strains indicating that PAC can manipulate/suppress the plant immune response [25]. Although the precise mechanisms how *P. subalpina* suppresses host induced defense mechanisms are unknown, effectors such as small secreted proteins (SSPs) predicted in the genome of *P. subalpina* may be candidates as they were shown to function as effectors in plant-fungal interactions [70, 75] and genome-wide differential gene expression studies during host colonization will help to identify possible effectors.

Beside the pathogen-related gene repertoire, our analysis shows that *P. subalpina* has also affinities with saprophyte-specific genes indicating that the species includes the signature of both lifestyles in the genome. A very similar positioning in the analysis was observed for *F. oxysporum*. Indeed, *F. oxysporum* not only includes the >70 pathogenic variants but non-pathogenic strains of *F. oxysporum* were also isolated as endophytes from asymptomatic roots [76–78].

Both species share a high number of β-lactamase/β-lactamase-related genes with saprophytes involved in the detoxification of β-lactam antibiotics. β-lactamases are well known fungal defense effector proteins [79], and detoxifying β-lactam antibiotics help to persist against antagonists. Besides the β-lactamase genes, those coding for sulfatases and different hydrolases were also enriched in saprophytic species including *P. subalpina* and *F. oxysporum*. In contrast to most pathogenic species, *P. subalpina* showed also an enlarged repertoire of PCWDEs involved in cellulose and hemicellulose breakdown as well as proteins with auxiliary activities supposed to be involved in lignin breakdown. Indeed, a strain of *P. fortinii* s.l. was shown to cause soft rot in autoclaved wood of beech and conifer species indicating that members of the PAC can degrade lignin, cellulose and hemicellulose [1].

### Why does P. subalpina stand out of the crowd?

The answer of two fundamental questions is still pending: (i) why are PAC species so amazingly successful in colonizing their hosts and (ii) does the host also benefit from the colonization by PAC. At least the second question may be answered by the genome sequence of *P. subalpina*.

Although often hypothesized, endophytic fungi of forest trees were rarely shown to be mutualistic for their hosts [4]. However, the closely related needle endophyte *P. scopiformis* was shown to produce rugulosin, a potent secondary metabolite against herbivory [80, 81]. Similarly, interaction studies using pathogens (*Phytophthora plurivora* and *Elongisporangium undulatum*), *P. subalpina* strains and *P. abies* seedlings showed that some of the *P. subalpina* strains effectively reduced mortality and disease intensity caused by the pathogens [82]. In addition, secondary metabolites were identified in PAC species that inhibited *Phytophthora* spp. [82], and the genome of *P. subalpina* encodes a high number of secondary metabolite key enzymes. Some of these are compatible with known pathways and products, such as melanin or ferrichrome-like siderophores. For example, enzymes PAC_05248 and PAC_13158 resemble SidC-like (=type II) and NPS1-like (=type IV) synthetases, i.e., two distinct types of ferrichrome-like siderophore-producing enzymes [83]. Also, two non-reducing type I PKSs of *P. subalpina* flanked by hydroxynaphthalene reductases likely involved in the melanin synthesis were recognized. Whereas gene PAC_1135 was placed in the same putative orthologous gene cluster as PKS1 of *G. lozoyensis*, gene PAC_07895 was placed in a second orthologous gene cluster and showed high similarities with the *alm* gene of *A. alternata*/the *PksP/Alb1* gene of *A. fumigatus*. All three proteins were experimentally shown to be involved in the melanin production [84–86]. Several Leotiomycete species included genes in both clusters (i.e. *B. cinerea*, *S. slcerotiorum*, *M. brunnea*), whereas melanized non-helotialean species and *G. lozoyensis* were included in one of the clusters (i.e. *C. geophilum*, *O. maius* and *C.globosum*). Moreover, no gene models of non-melanized species were included in one of these two clusters. To the best of our knowledge this is the first report for the duplication of the melanin pathway in ascomycetous fungi, although the redundancy of important secondary metabolite genes was reported previously [87, 88]. Still, the products of many of the secondary metabolite key enzymes and clusters remain unresolved. However, they may represent the chemical language of *P. subalpina* to interact via small molecules with host plants and other microorganisms in the rhizosphere.

Although the biomass and turnover rates of fine roots in forest ecosystems largely depend upon tree species, age of forest stands and climate, estimates indicate that as much as 30% of the net primary production is used for fine root production [89]. In an *Abies alba* stand for example >6.0 t/ha fine roots biomass with a cumulative length of > 20,000 km/ha was estimated [90] showing the importance of fine roots as carbon source. Given the fact that species of the *P. fortinii* s.l. *– A. applanata* species complex dominate many of the endophytic assemblages in fine roots of temperate and boreal forests [10], PAC species have access to a very substantial carbon source. *P. subalpina* can already colonize the future carbon source by entering healthy fine roots and may then switch to the saprophytic lifestyle as soon as the roots die off. Indeed, the signature of both lifestyles was observed in the genome although studies about the importance of PAC species in the fine root turnover are missing. Moreover, the fungus can escape the highly competitive soil community by colonizing the roots [91].

## Conclusions

The analysis of a globally distributed root endophyte allowed for detailed insights in the gene inventory and genome organization of a yet largely neglected group of organisms. Our analysis showed that the genome of *P. subalpina* has a versatile genome including genes for both a pathogenic and saprophytic lifestyle but showed also some affinities with ectomycorrhizal species. The degree of pathogenicity among strains of *P. subalpina* is high, as observed in *F. oxysporum*. In *F. oxysporum* pathogenicity is driven by mobile pathogenicity chromosomes [56]. Re-sequencing of multiple strains of *P. subalpina* will help identify the molecular basis of its pathogenicity. In addition, one central question will be to understand the evolutionary trajectory of PAC, i.e.whether PAC will become more pathogenic in future which would have a severe effect on forest ecosystems health, or whether the PAC-host interaction gets less antagonistic.

## Methods

### Selection of Phialocephala subalpina strain and DNA isolation

Strain UAMH 11012 (UAMH Centre for Global Microfungal Biodiversity, Toronto, Canada) was used for genome sequencing. The strain was originally isolated as single hyphal tip culture from *P. abies* fine roots in an undisturbed forest in Switzerland [14] and was classified using multiple classes of molecular markers [16, 38, 92]. The strain was grown in malt extract broth (50 ml 2% (w/v)) for 10 days at 20 °C under constant shaking. Mycelium was harvested by filtration, lyophilized, and total DNA was isolated using a CTAB-based protocol [93]. Strain identity was verified using microsatellite analysis [94] before the genome sequencing.

### Sequencing, assembly and gap closing

A whole genome shotgun strategy (WGS) on the Roche/454 GS FLX (454) was used and sequencing was performed at the Functional Genomic Centre of University Zürich/ETH Zürich (FGCZ). In total, 1.3 Mio shotgun reads as well as 2.3 Mio reads from one 3 kb paired-end library, 6.2 Mio reads of three 8 kb paired-end libraries and 309,909 reads of a 20 kb library were included in the assembly. The assembly was performed using newbler 2.5 with a minimum overlap of 50 bases and 98% sequence similarity. We noticed that newbler tends to open gaps due to the high number of pair-end reads in the assembly. Therefore, reads mapping to both sides of the gaps were identified and gaps were closed after manual validation.

### 454 sequencing of a normalized EST library

The same strain was used to generate a normalized EST library. The fungus was pre-cultivated in 50 ml of 2% malt broth (20 g l^-1^ malt extract; Difco) for 14 days at 20 °C under constant shaking. Then, the mycelium was homogenized with a blender for 30 s and 5 ml of the homogenized mycelium was transferred to new 50 ml of 2% malt broth (20 g l^-1^ malt extract; Hefe Schweiz). After 48 h the actively growing mycelium was harvested and immediately frozen in liquid nitrogen. Total RNA was isolated from approx. 75 mg fresh mycelium using the RNeasy plant mini kit (Qiagen, Hombrechtikon, Switzerland). Full-length cDNA was synthesized using the MINT kit (evrogen, Moscow, Russia) with a degenerated poly-T primer (5′-AAGCAGTGGTATCAACGCAGAGTAC (T)_4_G(T)_9_C(T)_10_VN-3′) during the first strand cDNA synthesis [95] and polTM1 (5′-AAGCAGTGGTATCAACGCAGAGTACTTTTGTCTTTTGTTCTGTTTCTTTTVN-3′) for the generation of dsDNA. The cDNA was normalized using the TRIMMER kit (evrogen) and the library was sequenced on the 454. Resulting reads were filtered for chimeras and then a whole transcriptome assembly was performed in newbler 2.3 with a minimum overlap of 50 bases and 98% sequence similarity.

### Repeat library construction

A repeat library was constructed based on the final assembly of the genome (see Additional file 8). In brief, putative repeat sequences were derived from RepeatScout analysis [96]. Low-complexity sequences and microsatellites were removed using the default filtering options in RepeatScout. In addition, sequences <50 bases and with <10 hit on the genome were excluded from further analysis. The remaining sequences were clustered using blastclust (-S 90 –L 0.9 –b F –p F) and only one sequence per cluster was used for further analysis. Blastx was used to exclude any sequences in the library not belonging to TEs (i.e. HET domain containing proteins or ubiquitin-like proteins). This draft library was then mapped against the genome using RepeatMasker and consensus sequences of complete TEs were derived. Finally, sequences were classified according to the systematic of Wicker et al. [97]. The manually curated TE library was used for genome masking before annotation.

### Annotation of the P. subalpina genome

The annotation strategy is presented in Additional file 9. In brief, a reference dataset of 1,089 gene models/proteins covered by full-length EST sequences was established and used as training dataset for Augustus [98]. *Ab-initio* gene prediction was performed on the masked genome using GeneMark-ES [99], Augustus [100] and FGENESH (*Neurospora* and *Ustilago* matrices). GenomeThreader [101] was used to calculate spliced alignments for *P. subalpina* ESTs and protein data from related fungal species. The program was run based on a *P. subalpina* specific splicing model trained using 454 EST dataset with the software BSSM4GSQ [102]. For the training of the splicing model, ESTs were chosen that showed a high coverage >98%, a sequence similarity of 100% and had only one hit on the genome resulting in >4,000 intron/exon junctions.

A total of 28,092 assembled ESTs as well as 27,819 non-assembled but well matching 454 singleton reads were mapped. Protein sequences of *Rhychnosporium secalis*, *S. sclerotiorum*, *B. cinerea*, *F. graminareum*, *N. crassa*, *Saccharomyces cerevisiae* were mapped using GenomeThreader. Finally, Jigsaw [103] was trained based on the 1,089 high confident gene models and used to calculate the best gene model using all the available evidence (predictors, ESTs, and trans-alignments). Subsequently, all gene models were manually curated in Apollo [104] and functional annotation was performed in PEDANT [105].

### Estimating the completeness of the P. subalpina genome and classification of gene models

The completeness of the *P. subalpina* genome and annotation was assessed by mapping two separate highly conserved core gene sets including 248 and 246 proteins respectively [106, 107]. In addition, the fraction of successfully and non-mapped ESTs was analyzed.

Gene models were classified based on the identity against the best hit in the Similarity Matrix of Proteins database (SIMAP) [39]. Proteins with identities ≥30% were considered as confidential gene prediction. The quality of the low identity genes (proteins with <30% identity with a protein sequence in SIMAP) was assessed by mapping the protein sequences against the assembled 454 EST dataset using GenomeThreader and analyzing the coverage of the gene model by ESTs. In addition, RNA-Seq data was available following the genome sequencing/annotation [108] (http://www.ebi.ac.uk/ena/data/view/PRJEB12610) was mapped against the genome using tophat (http://ccb.jhu.edu/software/tophat/index.shtml), discarding reads with bad mapping quality. Read coverage was calculated using coverageBed (http://bedtools.readthedocs.org/en/latest/content/tools/coverage.html) for each exon within the coding sequence. The coverage graph coverage graph was calculated in R using the ggplot2 package [109].

### Presence of RNAi pathway and analysis for the presence of RIP mechanism

A reference dataset of key proteins in the RNAi pathway of *Neurospora crassa* (ARGONAUT, DICER, and RNA-dependent RNA polymerases (RdRP)) was used to search for similar genes in *P. subalpina* as described in Laurie et al. [110]. In addition, the presence of repeat-induced point mutations (RIP) in the genome of *P. subalpina* was analyzed. As RIP results in C-to-T transitions at repetitive loci [45, 111] we analyzed di-nucleotide abundances of all predicted interspersed repeats and of non-repetitive control sequences using RIPCAL [112]. Overrepresented dinucleotides were identified by determining the fold change of the dinucleotide abundance between repeats and controls. *S. sclerotiorum* was included as a reference in the RIP analysis. Gene, repeat, and GC content were calculated using a sliding window analysis (window size: 1,000 bp, step size: 1,000 bp) and plotted for each scaffold. In addition, the genome of *P. subalpina* was mined for cytosine DNA methyltransferase genes of the Dnmt1 family involved in RIP and classified as described in Amselem et al. [54].

### Analysis of horizontal gene transfer from non-fungal species

A total of 163 proteins showed the best hit with non-fungal taxa (124 with bacteria, 20 with plants and 17 with metazoa) when mapped against the SIMAP database in PEDANT. The possibility of HGT for these genes was evaluated. In a first step, a BLAST search against the nr database (query coverage ≥40%; identities ≥20%; best 1,000 hits) was done and the taxonomic distribution and similarities of the hits were analyzed in R. Genes for HGT were selected as candidates if (i) they showed a biased taxonomic distribution of the hits (<15% fungal hits) and/or (ii) the fungal hits showed smaller bit scores in blast searches than non-fungal hits. Candidate genes were further analyzed using a phylogenetic approach. The full protein datasets of the ≤1000 best blastp hits against nr database for each candidate protein were downloaded. In addition, each candidate gene was also blasted against the genome data of phylogenetically closely related fungal genomes (*G. lozoyensis*, *B. cinerea*, *M. brunnea*, *S. sclerotiorum*). Protein sequences for each candidate gene were clustered with USEARCH v8.0.1517 (http://www.drive5.com/usearch/) [113] using the -cluster_fast option at an identity threshold of 0.95 (pre-sorted by length). If the resulting number of clusters exceeded 40, the threshold was sequentially reduced by 0.05 until cluster numbers were ≤ 40, or the threshold reached 0.5. This procedure was only applied to non-fungal sequences. Fungal BLAST hits were clustered at 0.95 if the number of clusters was ≤ 40. If not, the threshold was adjusted as described. The cluster representative sequence of each cluster was used for phylogenetic analysis. Protein sequences including the *P. subalpina* sequence were aligned using MAFFT [114] (E-INS-i method) and the alignment trimmed with TrimAl (http://trimal.cgenomics.org/trimal) [115] using the –strict setting. Maximum likelihood phylogenetic trees were inferred using FastTree 2.1 (http://meta.microbesonline.org/fasttree/) [116] with default settings. All trees were deposited in TreeBase (http://purl.org/phylo/treebase/phylows/study/TB2:S20196). The DNA-dependent RNA polymerase II (*RPB2*) sequence that is routinely used in phylogenetic studies was used as control. Due to its high conservation, only proteins with > 75% identity were included and clustering done at a 90% threshold.

### Secondary metabolism

Key proteins involved in the secondary metabolism of *P. subalpina* were searched by using conserved InterPro motifs of polyketide synthases (PKSs), non-ribosomal peptide synthetases (NRPSs) and related enzymes such as terpene synthases (TPC), and dimethyl allyl tryptophan synthases (DMATSs) followed by manual inspection of the genes to verify the domain arrangement in the case of PKSs and NRPSs. In addition, putative secondary metabolite clusters were identified by searching for genes encoding tailoring enzymes (e.g., acyl-, and methyltransferases, oxidoreductases, cytochrome P450 enzymes) up- and downstream of genes for key enzymes and searching for and promotors [117].

Gene cluster involved in melanin synthesis were predicted using protein (i) sequences of PKS genes experimentally shown to be involved in melanin synthesis such as *G. lozoyensis PKS1* (AAN59953.1; [85]), *Alternaria alternate alm* (BAK64048.1; [84, 118]) and *Alb1/PksP* of *Aspergillus fumigatus* (XP_756095.1; [86]), (ii) additional genes involved in the melanin synthesis pathway such as scytalone dehydratases (BC1G_144888) and hydroxynaphthalene reductases (BC1G_04230) [55] and (iii) by comparing orthologous gene clusters including candidate PKS derived from QuartetS analysis for the 14 ascomycetous species with the presence of melanization in the respective species. In addition, the NRPS genes putatively involved in siderophore synthesis were annotated.

### Comparative genome analysis

Comparative genome analyses were performed against a selection of published genomes of species showing different lifestyles (i.e. saprophytes, bio- and necrotrophic parasites and mycorrhizal species, Table 1). Special emphasis was put on selecting ascomycetous genomes and, whereever possible, genomes that are closely related to *P. subalpina*. All genomes used for comparative analysis were functionally annotated in PEDANT.

Putative orthologous gene clusters were analyzed using QuartetS [119] and the total number of clusters in which a specific species was present as well as pairwise shared clusters was recorded. Orthologous gene clusters enriched in pathogenic species were searched (four out of the five pathogenic species show entries for the respective cluster and ≤1 species of the six saprotroph species and ≤1 species of the two mycorrhizal species are present in the clusters respectively). Gene clusters enriched in saprophytes were searched using the same strategy. Both matrices were then subjected to principle component analysis using the vegan package in R [120] to analyze the position of *P. subalpina* compared to the other species. Moreover, FunCat terms [121] were enriched for these lifestyle enriched clusters by mapping *P. subalpina* geneIDs against the FunCat annotations and selecting the ten most frequent FunCat terms (any geneID/FunCat category was only considered once). In addition, putative orthologous gene clusters shared among the two mycorrhizal species *T. melanosporum* and *C. geophilum* with *O. maius* and *P. subalpina* was analyzed separately as the limited number of mycorrhizal species did not allow to properly defining lifestyle enriched clusters.

In a second step, the non-redundant portion of InterPro accessions per gene model, i.e. counting each InterPro accession per gene model only once, was analyzed. Besides some general statistics such as the number of distinct InterPro accessions within a species or different lifestyles or the total number of InterPro accessions, the number of significantly over- or underrepresented InterPro accessions in *P. subalpina* was determined by comparing their abundance in *P. subalpina* against the average observed in the 13 genomes using a Z-test [122]. Significantly over- and underrepresented InterPro accessions were then mapped against the gene ontology annotations (http://www.ebi.ac.uk/interpro/download.html) and enriched for GO terms. Moreover, InterPro accessions >2x overrepresented in pathogenic species and saprophytic species were mined and the resulting matrices were subjected to principle component analysis as described above.

In a third step carbohydrate-active enzymes were annotated using the CAZyme expert annotation pipeline [123] and the number of enzymes in the diverse CAZyme families were compared with a special emphasizes on the CAZyme families likely involved in cellulose (GH6, GH7, GH45), hemicellulose (GH10, GH11, GH26, GH31, GH67, GH115, GH134), pectin (GH28, GH53, GH78, GH79, GH88, GH105, GH106, GH127, PL1, PL3, PL4, PL9, PL11, CE8, CE12), and cutin layer breakdown (CE5). Moreover, a fourth category of CAZyme families were included that likely act on different of the above mentioned substrates (GH12, GH30, GH43, GH5, GH51, GH54, GH62, GH74, GH93). The total number of CAZYme modules per substrate class and species were calculated and subjected to principal component analysis as described above. In addition, proteins with auxiliary activities (AAs) that are hypothesized to be involved in the degradation of lignin were analyzed.

## Additional files

Additional file 1: Mapping statics for the assembled 454 ESTs. (PDF 197 kb)
Additional file 2: Validation of low identity gene models. (PDF 190 kb)
Additional file 3: Putative secondary metabolite clusters identified in the *P. subalpina* genome (XLSX 12 kb)
Additional file 4: General statistics on the presence of InterPro accessions in the analyzed genomes. (XLSX 10 kb)
Additional file 5: CAZyme modules related to PCWD used for principle component analysis. (XLSX 12 kb)
Additional file 6: General genome statistics for the species included in the present study. (XLSX 15 kb)
Additional file 7: PCA analysis based on InterPro accessions. Placement of the 13 ascomycete species and *P. subalpina* in PCA based on InterPro accessions described in Soanes et al. 2008 to be enriched in pathogens. (PDF 359 kb)
Additional file 8: Strategy to construct the manually-curated repeat library. (PDF 114 kb)
Additional file 9: Overview of the annotation strategy applied. (PDF 120 kb)

## Abbreviations

CAZyme: Carbohydrate-active enzymes
HGT: Horizontal gene transfer
PAC: *Phialocephala fortinii* s.l. – *Acephala applanata* species complex
PCWDE: Plant cell wall degrading enzymes
TE: Transposable elements
