# Supplementary figures and images for: Globally distributed root endophyte Phialocephala subalpina links pathogenic and saprophytic lifestyles
Source: BMC Genomics. 2016 Dec 9;17:1015. doi: 10.1186/s12864-016-3369-8 (PMC5148876; doi:10.1186/s12864-016-3369-8)

A

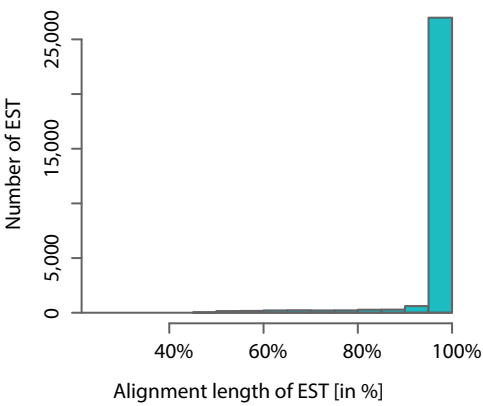

B

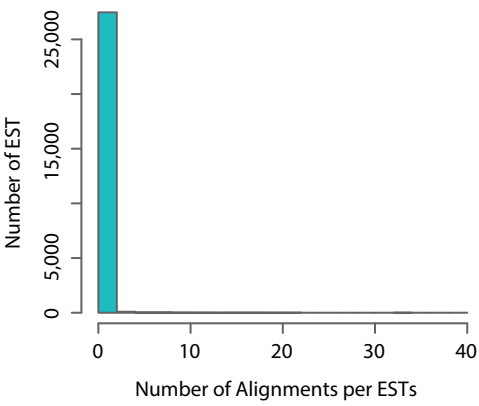

Supplement: Additional file 1: — Mapping statics for the assembled 454 ESTs. (PDF 197 kb) [file 12864_2016_3369_MOESM1_ESM.pdf]

A

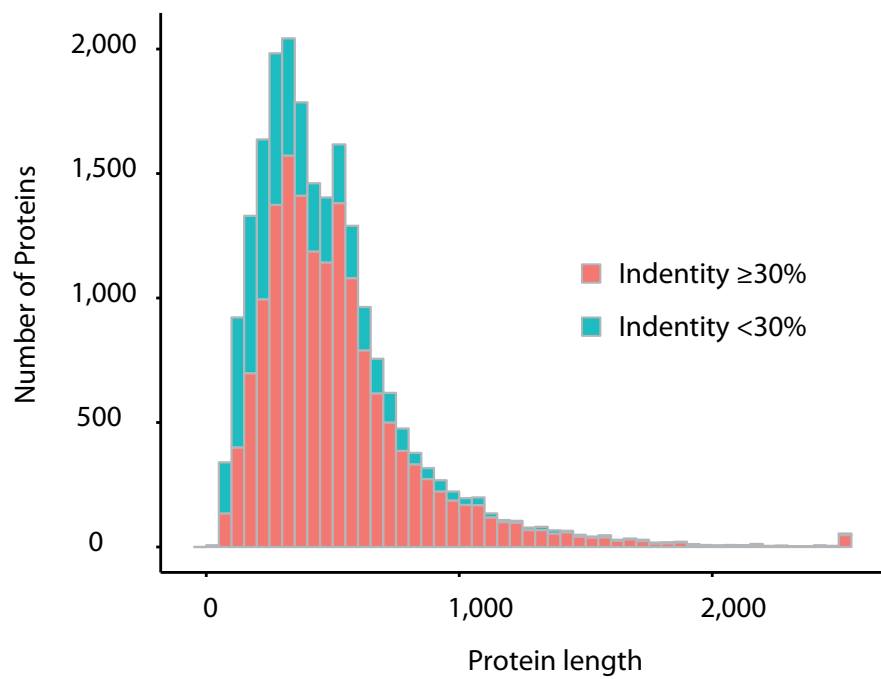

B

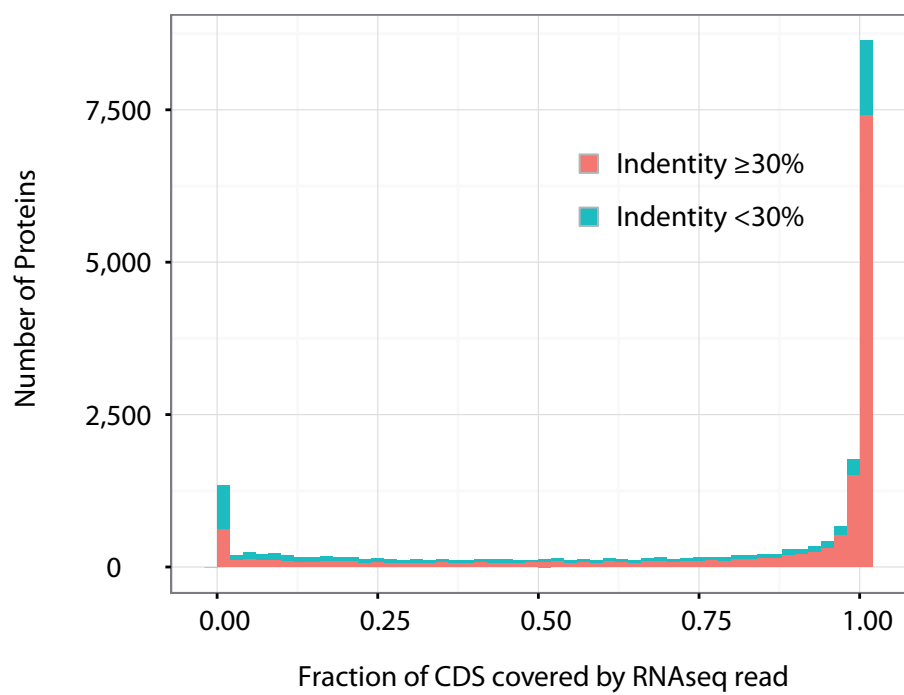

Supplement: Additional file 2: — Validation of low identity gene models. (PDF 190 kb) [file 12864_2016_3369_MOESM2_ESM.pdf]

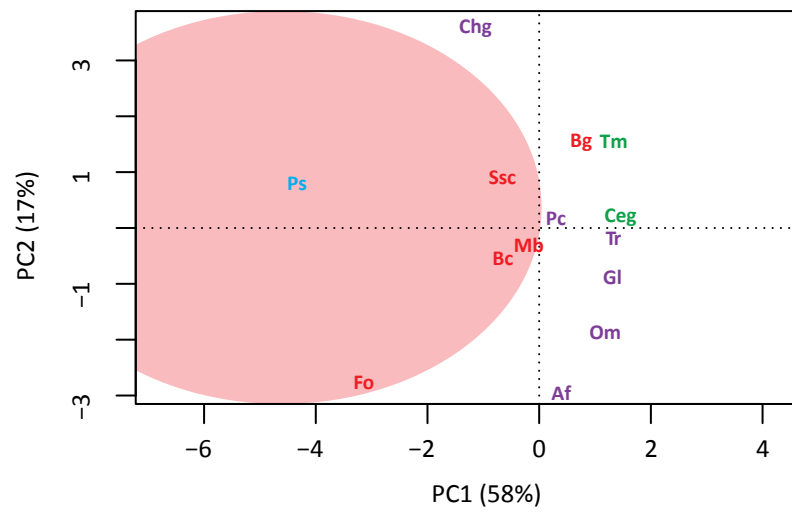

Supplement: Additional file 7: — PCA analysis based on InterPro accessions. Placement of the 13 ascomycete species and P. subalpina in PCA based on InterPro accessions described in Soanes et al. 2008 to be enriched in pathogens. (PDF 359 kb) [file 12864_2016_3369_MOESM7_ESM.pdf]
