# Supplementary material for: Globally distributed root endophyte Phialocephala subalpina links pathogenic and saprophytic lifestyles
Source: BMC Genomics. 2016 Dec 9;17:1015. doi: 10.1186/s12864-016-3369-8 (PMC5148876; doi:10.1186/s12864-016-3369-8)
Supplement: Additional file 8: — Strategy to construct the manually-curated repeat library. (PDF 114 kb) [file 12864_2016_3369_MOESM8_ESM.pdf]

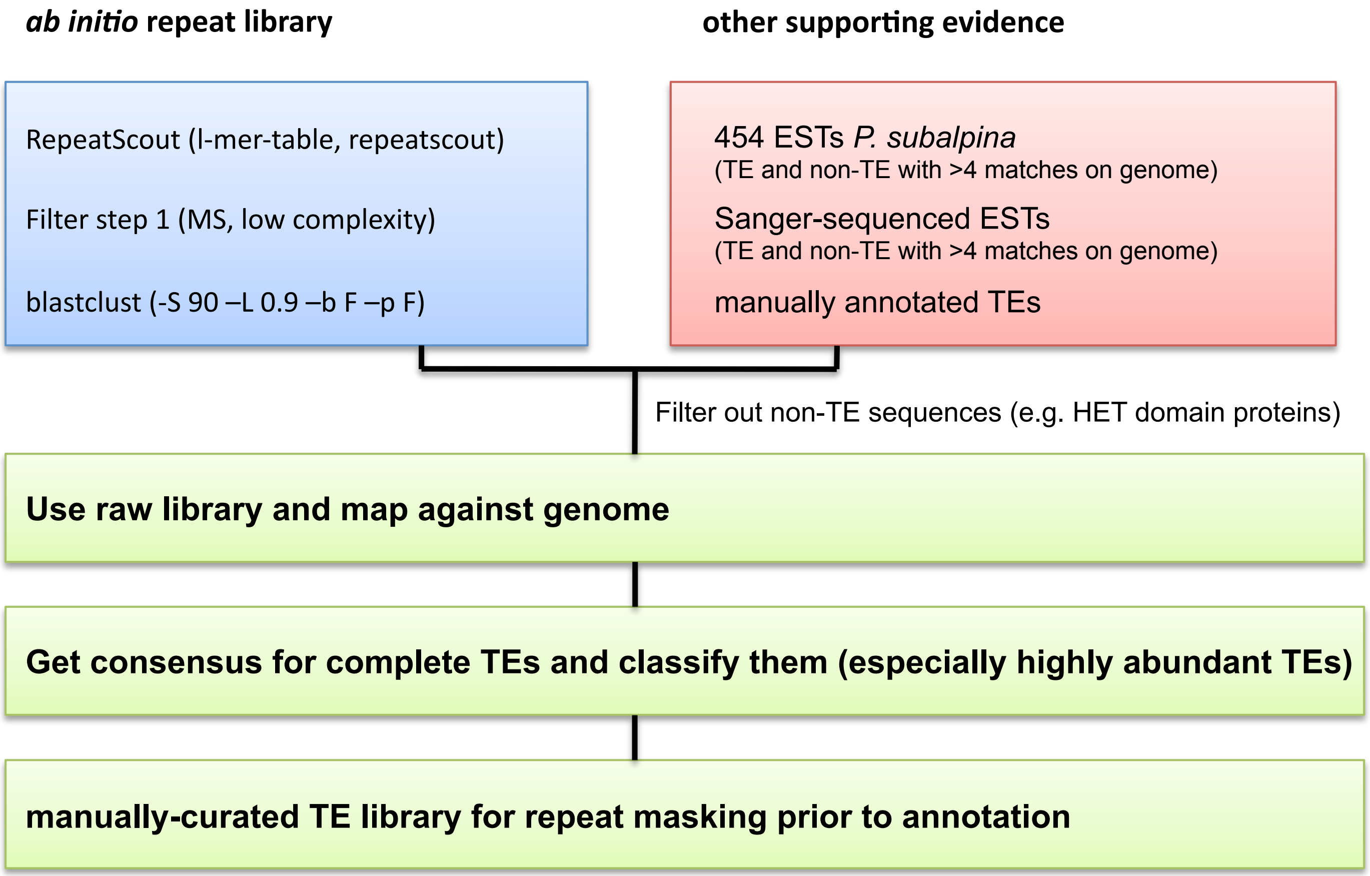
ab initio repeat library
other supporting evidence
RepeatScout (l-mer-table, repeatscout)
Filter step 1 (MS, low complexity)
blastclust (-S 90 –L 0.9 –b F –p F)
454 ESTs P. subalpina
(TE and non-TE with >4 matches on genome)
Sanger-sequenced ESTs
(TE and non-TE with >4 matches on genome)
manually annotated TEs
Filter out non-TE sequences (e.g. HET domain proteins)
Use raw library and map against genome
Get consensus for complete TEs and classify them (especially highly abundant TEs)
manually-curated TE library for repeat masking prior to annotation
