# Supplementary material for: Globally distributed root endophyte Phialocephala subalpina links pathogenic and saprophytic lifestyles
Source: BMC Genomics. 2016 Dec 9;17:1015. doi: 10.1186/s12864-016-3369-8 (PMC5148876; doi:10.1186/s12864-016-3369-8)
Supplement: Additional file 9: — Overview of the annotation strategy applied. (PDF 120 kb) [file 12864_2016_3369_MOESM9_ESM.pdf]

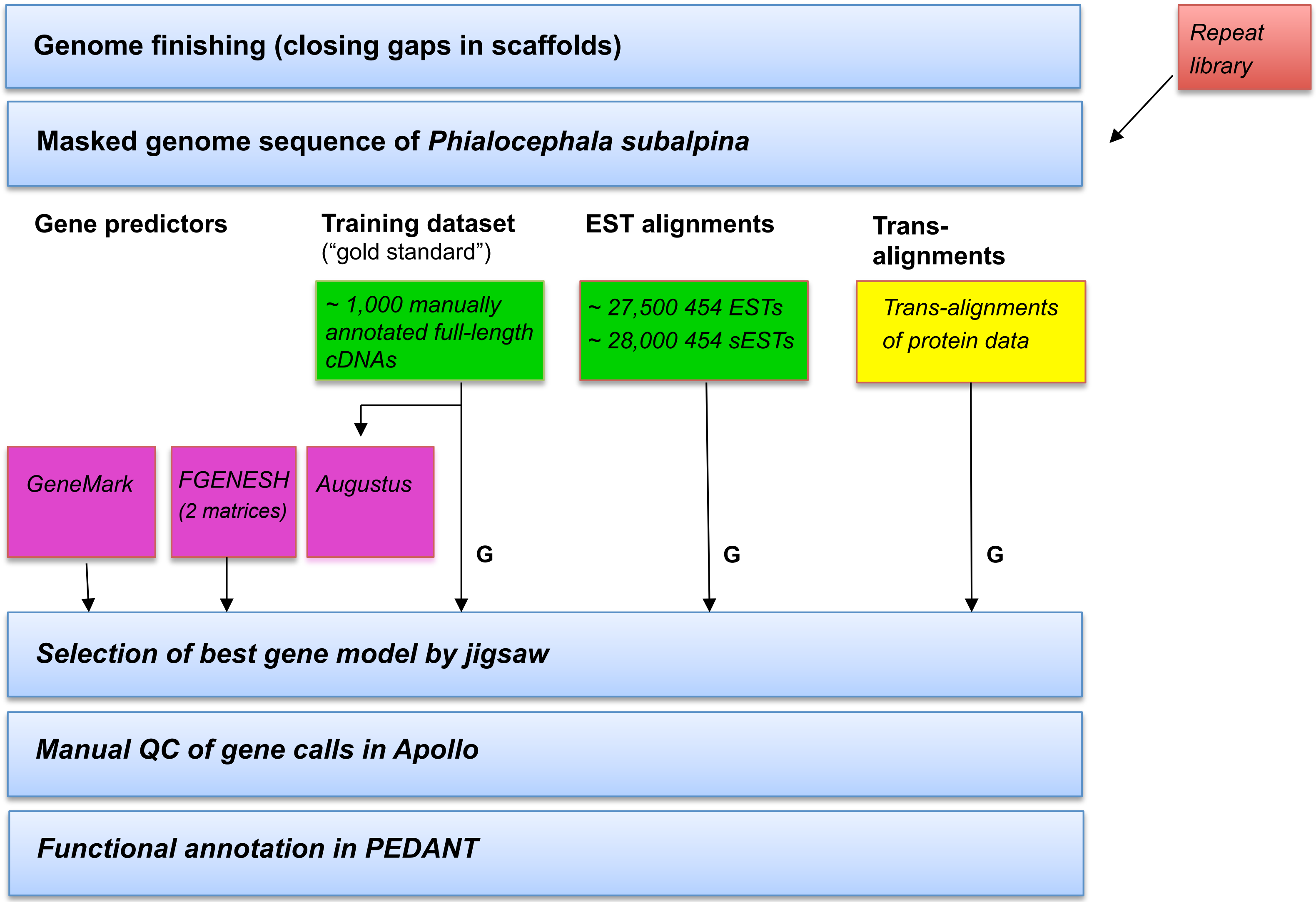

G = GenomeThreader (spliced alignments of ESTs, cDNA and proteins using splice site models trained for *P. subalpina*)
